# Magnetic bioassembly platforms towards the generation of extracellular vesicles from human salivary gland functional organoids for epithelial repair

**DOI:** 10.1016/j.bioactmat.2022.02.007

**Published:** 2022-02-16

**Authors:** Ajjima Chansaenroj, Christabella Adine, Sawanya Charoenlappanit, Sittiruk Roytrakul, Ladawan Sariya, Thanaphum Osathanon, Sasitorn Rungarunlert, Ganokon Urkasemsin, Risa Chaisuparat, Supansa Yodmuang, Glauco R. Souza, João N. Ferreira

**Affiliations:** a Avatar Biotechnologies for Oral Health and Healthy Longevity Research Unit, Faculty of Dentistry, Chulalongkorn University, Bangkok, 10330, Thailand; b Faculty of Dentistry, National University of Singapore, 119077, Singapore, Singapore; c Department of Biomedical Engineering, Faculty of Engineering, National University of Singapore, 119077, Singapore, Singapore; d Functional Ingredients and Food Innovation Research Group, National Center for Genetic Engineering and Biotechnology, National Science and Technology Development Agency, Pathum Thani, 12120, Thailand; e The Monitoring and Surveillance Center for Zoonotic Diseases in Wildlife and Exotic Animals, Faculty of Veterinary Science, Mahidol University, Nakhon Pathom, 73170, Thailand; f Department of Anatomy, Faculty of Dentistry, Chulalongkorn University, Bangkok, 10330, Thailand; g Department of Preclinical and Applied Animal Science, Faculty of Veterinary Science, Mahidol University, Nakhon Pathom, 73170, Thailand; h Research Affairs, Faculty of Medicine, Chulalongkorn University, Bangkok, 10330, Thailand; i University of Texas Health Sciences Center at Houston, Houston, TX, 77030, USA; j Nano3D Biosciences Inc., Houston, TX, 77030, USA; k Greiner Bio-One North America Inc, Monroe, NC, 28110, USA

**Keywords:** Salivary gland, Hyposalivation, Human dental pulp stem cells, Magnetic bioassembly, Organoids, Exosome

## Abstract

Salivary glands (SG) are exocrine organs with secretory units commonly injured by radiotherapy. Bio-engineered organoids and extracellular vesicles (EV) are currently under investigation as potential strategies for SG repair. Herein, three-dimensional (3D) cultures of SG functional organoids (SGo) and human dental pulp stem cells (hDPSC) were generated by magnetic 3D bioassembly (M3DB) platforms. Fibroblast growth factor 10 (FGF10) was used to enrich the SGo in secretory epithelial units. After 11 culture days via M3DB, SGo displayed SG-specific acinar epithelial units with functional properties upon neurostimulation. To consistently develop 3D hDPSC *in vitro*, 3 culture days were sufficient to maintain hDPSC undifferentiated genotype and phenotype for EV generation. EV isolation was performed via sequential centrifugation of the conditioned media of hDPSC and SGo cultures. EV were characterized by nanoparticle tracking analysis, electron microscopy and immunoblotting. EV were in the exosome range for hDPSC (diameter: 88.03 ± 15.60 nm) and for SGo (123.15 ± 63.06 nm). Upon *ex vivo* administration, exosomes derived from SGo significantly stimulated epithelial growth (up to 60%), mitosis, epithelial progenitors and neuronal growth in injured SG; however, such biological effects were less distinctive with the ones derived from hDPSC. Next, these exosome biological effects were investigated by proteomic arrays. Mass spectrometry profiling of SGo exosomes predicted that cellular growth, development and signaling was due to known and undocumented molecular targets downstream of FGF10. Semaphorins were identified as one of the novel targets requiring further investigations. Thus, M3DB platforms can generate exosomes with potential to ameliorate SG epithelial damage.

## Highlights

• Magnetic bioassembly platforms scale-up the production of salivary gland organoids.
• Exosomes from organoids rescued up to 60% of gland epithelial growth.
• Transplanted gland organoids from magnetic bioassembly platforms rescued 25% of gland epithelial growth.
• Exosomes from dental pulp stem cells in magnetic bioassembly platforms marginally alter epithelial growth.
• 99 proteins were differentially expressed in exosomes from organoids.

## Graphical abstract

**Figure:**
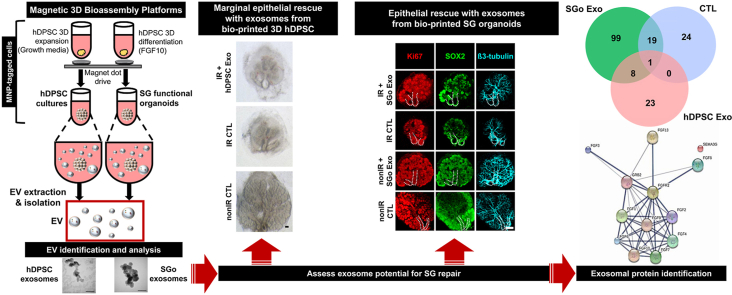

## 1 Introduction

Radiation therapy is a gold standard intervention for patients with late stage head and neck cancers, although often salivary glands (SG) lie on the radiation field causing epithelial damage to the saliva-producing secretory units [1]. As a result, there is a marked reduction in their saliva flow rate impacting daily oral routines in these cancer patients due to dry mouth, dysphagia, speaking difficulties, dental caries, oral mucositis, among others [2]. However, conventional therapies using palliative approaches have been mainly utilized for SG hypofunction. Thus, therapeutic strategies to repair or replace damaged or diseased SG secretory units or organs are essential [3]. Various organoid biofabrication platforms have been described over the past decade to ultimately aid on the organ regeneration efforts beyond organogenesis and novel drug discovery targets. Organoids can be derived from primary cells or multipotent stem cells as is the case of the seminal works with adult stem cells (ASCs) aiming for the development of small intestinal epithelial organoids that relies on a stem cell niche that rapidly self-renews in adult mammals [[4], [5], [6]]. After such groundbreaking reports, exocrine glands such as mammary gland [7,8], prostate [9,10] and the SG [[11], [12], [13]] were also generated by using a similar paradigm. However, several organoid platforms rely on the use of xeno-derived matrices like Matrigel [14] or Laminin gels [15], which face challenges in terms of clinical applicability. Therefore, our research group has biofabricated SG organoids without xeno-derived matrices or scaffolds [16,17]. Another concern in the SG organoid development is the tendency to lose the secretory phenotype in epithelial units throughout culture [18,19]. Our recent work with human dental pulp stem cells (hDPSC) addresses this issue with the supplementation of Fibroblast growth factor 10 (FGF10) and by providing a neuronal network to SG organoids [16]. FGF10 is a key signaling cue for SG morphogenesis [[20], [21], [22], [23]] and innervation is essential for a sustained neurotransmitter stimulation to the different areas/regions (central and peripheral) of the SG organoid that display epithelial compartments [16]. There is a strong evidence that such innervation can maintain the epithelial stem/progenitor cells (e.g. SOX2+ cells) that are crucial for repair after radiation injury [[23], [24], [25], [26]]. In our previous work, SG organoids were developed with a magnetic three-dimensional bioassembly (M3DB) platform, and such organoids displayed epithelial compartments that can stimulate epithelial and neuronal growth in the irradiated SG upon transplantation [16]. However, such therapeutic effects may arise from the paracrine activity of the secretome or extracellular vesicles (EV) cargo produced by mesenchymal-derived ASC [[27], [28], [29], [30]] which have similar characteristics as the hDPSC used in our SG organoid platform [16]. Hence, it would be relevant to assess the EV arising from both hDPSC and our SG organoids *in vitro* to understand how SG epithelial repair takes place as well as to optimize the SG organoid platform for an enhanced release of key EV paracrine cues.

Thus, the objectives of this study were: (1) to generate, isolate and identify EV in the conditioned media of hDPSC 3D cultures and SG organoids developed by M3DB; (2) to assess biological repair events after treatment of epithelial SG injuries with the above-mentioned EV; and (3) to identify novel signaling cues in EV that may optimize organoid development towards epithelial SG repair.

## 2 Materials and methods

### 2.1 Adult stem cell culture

hDPSC were obtained from AllCells (Alameda, CA, USA) and cultured in Dulbecco's Modified Eagle Medium (DMEM) Glutamax with 10% fetal bovine serum and 1% antibiotic-antimycotic. Media was changed on alternate days and hDPSC were passaged at ∼75–80% confluency. Flow cytometry (FC) was executed to detect hDPSC-specific surface markers. All culture reagents and chemicals were obtained from Thermo Fisher Scientific or its subsidiaries Gibco or Invitrogen (Waltham, MA, USA) and Sigma-Aldrich Pte. Ltd. (Singapore) unless otherwise indicated.

### 2.2 Characterization of hDPSC phenotype

hDPSC were subcultured and trypsinized using TrypLE. Trypan Blue was utilized to count the number of viable cells. hDPSC were assessed for doubling time up to passage 6 as previously described [16]. As a minimum 5 × 10^5^ cells/tube were used for FC analysis. Conjugated primary antibodies in Table S1 were incubated for 20 min at 4 °C. Four percent of paraformaldehyde (PFA) was used to fix the cells at room temperature (RT). Triton-X in MACS buffer (Miltenyi Biotec, Bergisch Gladbach, Germany) was used for cell permeabilization. For FC analysis, MACS buffer was applied to wash and resuspend the cells. Data analysis was run using LSR Fortessa™ cell analyzer (BD Bioscience, San Jose, CA, USA). and FlowJo software (Tree Star, Ashland, OR, USA). When building multicolor flow cytometry panels, fluorescence minus one controls were run to ensure that there was no spectral overlap between antibody markers and to set up the gates. Gating was done after staining with isotype IgG control (equal dilutions alike primary antibodies) or after analyzing unstained cells. hDPSC between passage 3 and 6 were used in subsequent experiments because the expression patterns of hDPSC, hMSC, pro-mitotic and SG progenitor markers were consistent.

### 2.3 Bioassembly hDPSC in 3D with M3DB platform

**Fig. 1:**
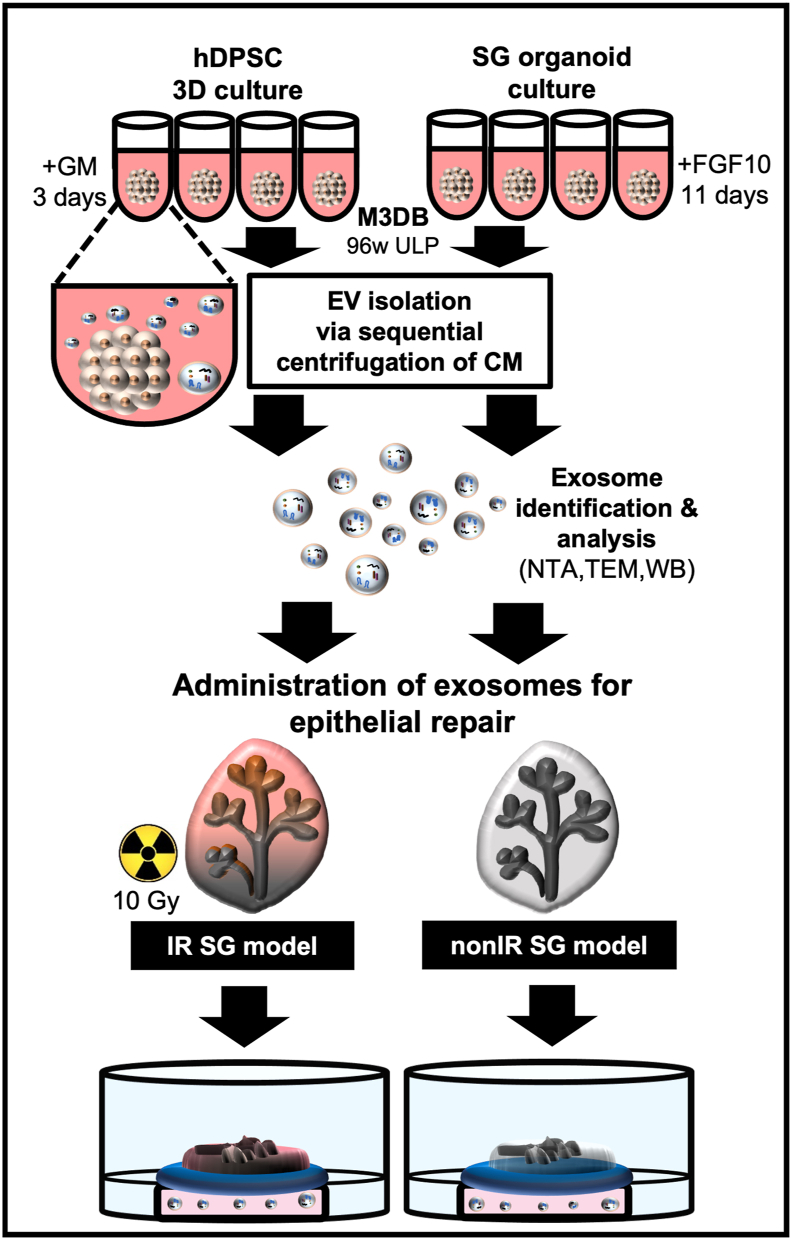
Flowchart with the exosome strategies for SG epithelial repair using 3D hDPSC cultures and SG organoids prepared via magnetic three-dimensional bioassembly (M3DB). hDPSC were magnetized in advance with nanoparticles and assembled with a magnetic drive for a 96-well ultra-low attachment plate (96w ULP) – see supplementary data in Fig. S1. The differentiation to SG organoids was carried out with media enriched with FGF10; while hDPSC were kept in growth media (GM). Sequential centrifugation steps were performed to collect all EV from secretome of conditioned media (CM) and identify exosomes. Magnetic bioassembly of hDPSC-derived and SG organoid-derived exosomes (100% extract) were then administered into SG growth media to treat epithelial repair in irradiated (IR) and non-irradiated (nonIR) SG models.

Three-dimensional (3D) spheroid formation was conducted using a solution of iron oxide cross‐linked with poly‐l‐lysine solution and gold, also abbreviated as MNP (Nanoshuttle™, Greiner Bio-one North America, Monroe NC, USA). The MNP solution is known to magnetize cells via electrostatic interactions with the cellular membranes during an overnight static incubation. hDPSC were assembled by M3DB based on the original protocol [16] (Fig. 1) and were previously optimized to generate controllable and consistent sphere-like 3D cultures using a combination of the following parameters: concentration of the MNP (Nanoshuttle™) and cell seeding density. Briefly, hDPSC were incubated during ∼12 h with MNP at a concentration of 2.5 × 10^4^ cells/1 μL MNP for cell magnetization. The magnetized hDPSC were enzymatically isolated by TrypLE and resuspended with growth medium prior to seeding them into ultra-low attachment 96-well plates (Corning, NY, USA) at 3 × 10^6^ cells per well. Next, magnetized hDPSC were centrifuged to aggregate cells at the bottom of each well. A magnetic drive with 96 neodymium magnet dots at 0.062500 OD was placed underneath the culture plates (Greiner Bio-one North America). This drive provides a fixed magnetism, and possesses a weak magnet with approximately 200 G at the bottom of Petri dishes, and the gradient is approximately 50 G/mm hDPSC were cultured for 3 days via M3DB and their conditioned media was collected for further extract and isolate EV (Fig. 1 and S1). A DMi8 fluorescent microscope (Leica Microsystems, Germany) was used to assess size and morphology of M3DB spheroids. Spheroids were imaged by a high-resolution Perfection V550 Epson flatbed scanner (Seiko Epson Corporation, Japan) to measure their diameter.

### 2.4 Epithelial differentiation towards SG organoids

Once hDPSC had reached >90% viable cells after 3 days over a magnetic drive, the SG epithelial differentiation stage was initiated to generate SG organoids. The epithelial differentiation media (EDM) supplemented with 400 ng/ml Fibroblast growth factor 10 (FGF10, Catalog No. 345-FG-025/CF, R&D Systems, Minneapolis, MN, USA) was used to culture the cells until day 11 (Figs. 1 and S1a) as optimized in our previous study [16]. Media was changed daily until day 8.

### 2.5 Whole-mount immunohistochemistry

Magnetic bioassembly of hDPSC and SG organoids (SGo) were fixed in 4% PFA, permeabilized with a nonionic surfactant and blocked with a PBS buffer with 10% donkey serum and 5% BSA. Then, primary antibodies (dilutions listed on Table S1) were added, incubated overnight with the 3D tissues at 4 °C, followed by incubation with secondary antibodies with Alexa Fluor 488, 594 and 647 fluorophore tags. Next tissues were counterstained with Hoechst 33342 and mounted on glass slides. Assembled 3D tissues were evaluated by using DMi8 fluorescent microscope (Leica Microsystems, Germany), Zeiss LSM 710 confocal microscope (Zeiss, Germany), and Leica TCS SP8 DM6000 CFS upright confocal with a two-photon laser-scanning microscope (Leica Microsystems).

Mouse salivary gland organs from EV treatments and SGo transplantation experiments were processed for immunohistochemistry as per aforementioned protocol with Ki67, SOX2 and β3-tubulin antibodies (dilutions listed on Table S1). Immunofluorescent labeled micrographs were evaluated and quantified (intensity, volume, area) by Imaris software (version 9.6, Oxford Instruments, Zurich, Switzerland). Quantification of the neuronal intensity was normalized to the overall area of the gland explant as demarcated by the nuclear stain (Hoechst 33342).

All primary antibodies utilized in this study were validated against control salivary gland tissue (either adult or embryonic) on a previous report [16]. Hence, to avoid repetitions and duplication of controls, the majority of those controls were not displayed herein.

### 2.6 Quantitative polymerase chain reaction

RNA was obtained from SGo and treated with DNAse by using Ambion MicroRNA kit as per manufacturer's protocol. Nanodrop ND1000 was used to evaluate total RNA concentration. The iScript® was used for cDNA synthesis. The qPCR was performed in a Bio-Rad CFX96 system. The reference gene in this study was *s29*. The 2^-(ddCT)^ calculation protocol was applied to evaluate the relative expression of target genes. The primer sequences are listed on Table S2.

### 2.7 Salivary amylase assay

EnzChek Ultra Amylase Assay Kit was performed to assess the activity of α-amylase. At the end of the SGo culture, the conditioned media was kept for measuring α-amylase secretion after neurotransmitter stimulation. The digestion of DQ™ starch substrate by the presumptive α-amylase was determined. The fluorescent signal was quantified at 495 nm by a microplate reader (Tecan Group Ltd, Männedorf, Switzerland). The standard curve was generated by starch standards. The experimental values were deducted from fresh media fluorescent values.

### 2.8 Intracellular calcium mobilization

To measure and visualize the calcium influx in phenol-free media, a Fluo-4 direct calcium assay was utilized as per the manufacturer's protocol. Addition of calcium chloride to the SGo culture provided a source of calcium ion. For the neurostimulation, 10–1000 μM Carbachol was added into the culture medium to stimulate presumptive muscarinic neurons for 18h. Then, SGo was cultured with 10–1000 μM Isoproterenol to stimulate adrenergic neurons. The time-lapse imaging (every 5 s per cycle) by a DMi8 fluorescence microscope (Leica, Germany) was used to determine the fluorescence intensity and intracellular calcium mobilization.

### 2.9 EV extraction, isolation and analysis

Extracellular vesicles (EV) from both the conditioned media of hDPSC cultures and SG organoids in M3DB platforms were extracted and isolated via the sequential centrifugation methodology [31,32] according to position statement by International Society for Extracellular Vesicles (ISEV) [32]. The EV from hDPSC and from SG organoids in M3DB platforms were analyzed after 11 days of culture, and a minimum of 800 μl of conditioned media was necessary. Next, isolated EV were diluted 10x and analyzed in the nanoparticle tracking analyzer (NTA) with NanoSight NS300 (Malvern Panalytical, Spectris, Malvern, UK) for size dimensions and distribution. For ultrastructural analysis, EV isolated from conditioned media were fixed with 2% PFA, 2% sucrose and 3% glutaraldehyde in PBS. In post fixation, exosome solutions were placed on the Formvar-carbon coated grids for 10–15 min. Stain with 1% uranyl acetate for up to 4 min. The exosome morphology was evaluated by JEOL JEM-1400 transmission electron microscope (JEOL USA, Inc., MA, USA) using 100 kV at 300,000X magnification. Immunoblotting was also performed to identify the exosome-specific protein (more details in section 2.12 for western blotting).

### 2.10 Administration of exosomes in SG organ models

Animal experimentation complied with the ARRIVE guidelines and was performed in accordance with the National Institutes of Health guide for the care and use of laboratory animals (NIH Publications No. 8023, revised 1978) under protocol number R14-0306 issued by the National University of Singapore Institutional Animal Care and Use Committee (NUS IACUC). Pregnant mice were sacrificed using CO_2_-based euthanasia followed by cervical dislocation. Submandibular and sublingual SG of embryonic day 13 ICR mice were isolated under M80 stereomicroscope (Leica, Germany). These explants were cultured on Whatman Nucleopore Track-etch membrane 0.2 μm porous membranes in 55-mm glass-bottom dishes (MatTek, Ashland, MA, USA) containing 200 μl of media. This culture medium was composed of DMEM/F12 supplemented with 50 μg/ml transferrin, 150 μg/ml vitamin C, 100 U/ml penicillin and 100 μg/ml streptomycin, which will be referred from here onwards as growth media (GM). Four to six glands were cultured in at 37 °C and 5% CO_2_. Eight hours later, fetal SG were given 10 Gy of ionizing radiation to develop the SG model damaged by irradiation (IR SG model) using a BIOBEAM 8000 (Gamma-Service Medical GmBH, Germany) [16,33]. Approximately 1–2 h after radiation, SGo were used as controls and transplanted and placed on top of the porous Whatman membranes, next to the ducts of the fetal glands. As for the EV, same concentration of exosome extracts (as determined by Lowry assay in section 2.11) from hDPSC and SGo were added to the GM under the fetal SG immediately after radiation. Gland explants were imaged (bright field) daily up to day 3 of culture using the M80 stereomicroscope (Leica, Germany) and the epithelial buds were counted at each timepoint using ImageJ (NIH, USA).

### 2.11 Exosomal protein cargo analysis by mass spectrometry

After EV/exosome isolation via the sequential centrifugation, EV solutions were treated with acetone overnight at −20 °C. The protein cargo was then centrifugated at 10,000 g for 15 min. The protein pellet was dried and stored at −80 °C prior to use. Total protein from EV solution was evaluated with a Lowry protein assay. Protein samples were subjected to in-solution digestion. The digestion process was performed as described [34]. The digested samples were protonated by using 0.1% formic acid and dried prior to injecting into the nano/capillary LC system. The LC-MS/MS was used to examine the peptides quantification from the digested samples as reported [34]. Each sample was run in triplicate. Peptides in samples were computed by MaxQuant 1.6.6.0 (Max-Planck-Institute of Biochemistry, Martinsried, Germany) using the Andromeda search engine to correlate MS/MS spectra to the UniprotKD knowledge database for *Homo sapiens* [35]. Seven amino acids were needed for protein detection. Only proteins with at least two peptides and at least one single peptide were counted as being detected and used for further analysis. Protein false discovery rate (FDR) was established at 1% and anticipated by using the reversed search sequences. The maximal number of modifications per peptide was set at 5. As a search FASTA file, the 173,260 proteins appear in the human proteome downloaded from UniprotKD on April 27, 2020. Potential contaminants displayed in the contaminants.fasta file were automatically placed into the search engine by the software. The MaxQuant ProteinGroups.txt file was uploaded to Perseus software version 1.6.5.0, potential contaminants were deleted from the file. Maximum intensities were log transformed. Missing values were assigned as zero in the Perseus software. The statistical analyses and visualization were performed using the MultiExperiment Viewer (MeV) in the TM4 suite software [36]. Biological processes and protein organization were examined through the Panther protein classification [37]. Venn diagrams were applied to demonstrate the differences among proteins that are differentially expressed in exosomes/EV from magnetic assembled SGo when compared to hDPSC-derived exosomes and a control group with secretome from hDPSC conditioned media without FGF10 [38]. General and predicted functional interaction networks among known proteins and other small molecules were investigated by STITCH database version 5.0 [39].

### 2.12 Western blotting

Tissue protein extraction reagent (T-PER™, Thermo Fisher Scientific) combined with phosphatase and protease inhibitors (Roche, Switzerland) were utilized. SGo were homogenized on ice by using an ultrasonic homogenizer (VCX 130, Sonics and Materials Inc., Newtown, CT, USA). Cell lysates were centrifugated at 15,000×*g* for 15 min at 4 °C. Supernatants were incubated at 100 °C for 5 min in Laemmli buffer. After peptide separation in SDS-PAGE, proteins were blotted to polyvinylidene difluoride (PVDF) membranes at 100 V for 1 h. Skim milk in Tris buffered saline (TBS) with Tween 20 were applied to block the membrane, and then incubated with either anti-KRT5, anti-KRT14, anti-α-SMA, and anti-GAPDH (antibody dilutions in Table S1). Bound antibodies were detected with HRP-linked antibodies. The Clarity Western ECL Substrate detection kit was used to develop the blot as per manufacturer protocol. Chemiluminescence was analyzed using Bio-Rad ChemiDoc MP Imaging System.

Protein extraction was performed from the SG organoid-derived and hDPSC-derived EV and quantified with bicinchoninic acid assay. Briefly, RIPA buffer supplemented with protease inhibitors were utilized to lyse the following: (1) EV derived from SG organoids supplemented with FGF10; (2) EV derived from hDPSC in M3DB; (3) EV derived from conditioned media from MCF7 cell cultures (positive control previously shown to exhibit EV [40]) offered by Dr. Muttarin Lothong. Protein content was concentrated by 10 kDa spin concentrators (GE Healthcare, Buckinghamshire, UK). For identification of exosome-specific markers and SEMA3G, protein lysates were denatured and incubated with either anti-ALIX, anti-TSG101 and anti-SEMA3G (dilutions in Table S1). The bound antibodies were detected with HRP-linked antibodies. The luminol detection kit was used to develop the blot and chemiluminescence was analyzed using Bio-Rad ChemiDoc™ Touch Imaging System. All equipment and chemicals are from Bio-Rad Laboratories, Inc. (Hercules, CA, USA) unless otherwise stated.

### 2.13 Statistical analysis

All statistical analyses and heat maps were carried out using GraphPad Prism version 6 or 8 (GraphPad software, San Diego, CA, USA). Unpaired Student's t-test was applied to analyze differences between two experimental groups. Differences through culture were compared by paired t-tests. One-way analysis of variance (ANOVA) followed by *Dunnett's* or *Tukey's post-hoc* tests for comparing values from 3 groups. Significance was set at *p* < 0.05.

## 3 Results

Bio-engineering steps were taken to assemble hDPSC and SG-like 3D cultures (organoids) via M3DB and isolate their EV for evaluating SG epithelial repair (Fig. 1).

### 3.1 M3DB maintained hDPSC genotype and phenotype after bioassembly

**Fig. 2:**
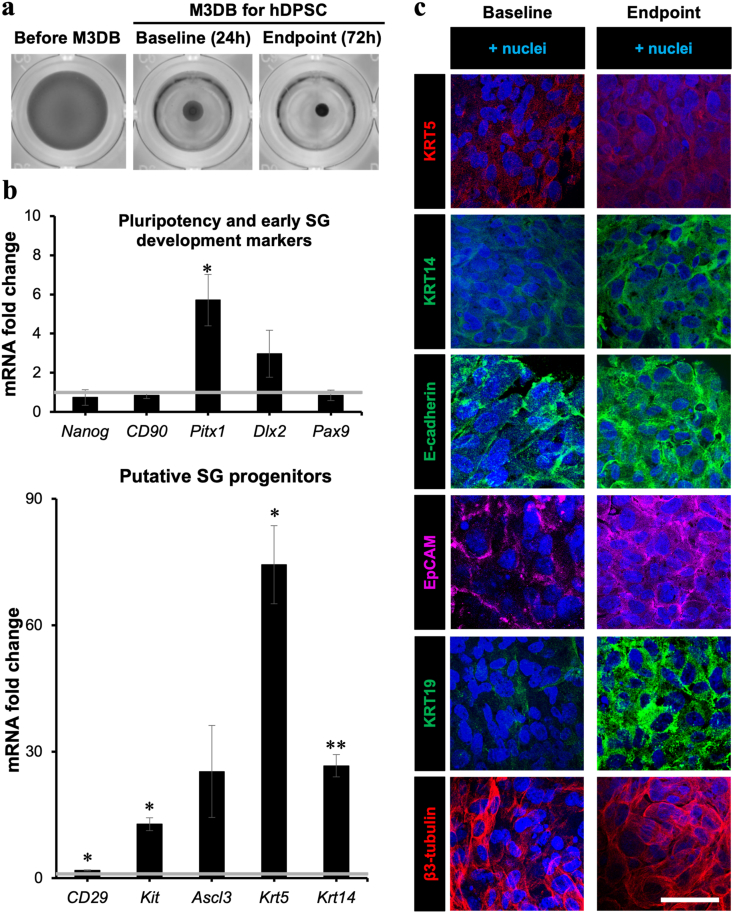
Genotypic and phenotypic characterization of hDPSC 3D bio-assembled cultures. (**a**) Digitally scanned micrographs of hDPSC cultures. (**b**) 3D hDPSC cultures via M3DB supported the maintenance of pluripotency, hDPSC and oral primordium genetic markers. Values on Y-axis represent mean fold change in gene expression by qPCR relative to undifferentiated hDPSC monolayer (baseline) and normalized to the housekeeping gene *Rps29.* Data are presented as mean ± SEM from *n* = 4. *p < 0.05 and **p < 0.01 when compared to baseline group using multiple Student's *t*-tests with 5% False Discovery Rate. Gray horizontal line represents baseline at mean RNA fold change of 1. (**c**) DPSC 3D cultures formed by M3DB expressed SG-specific cytokeratins markers in oral ectoderm, cell *adherens* junctions and neuronal. Representative spheroid images immuno-labeled with oral primordium progenitor markers (cytokeratins: KRT5, KRT14), ductal luminal (KRT19), pan-epithelial markers such as *adherens* junctions (E-cadherin and EpCAM) and neuronal (β3-tubulin). Images shown are maximum intensity projections. Scale bar: 50 μm.

The hDPSC population doubling time (PDT) was significantly different at passage 7 when compared to passage 1 and 2 (*p* < 0.05) (Fig. S2a). Flow cytometry histograms (Fig. S2b) revealed that hDPSC immunophenotype at passages 3–6 were strongly purified with human MSC surface markers (CD73 and CD105), and did not present hematopoietic stem cell surface markers (CD34 and CD45). Thus, this confirmed that hDPSC presented a mesenchymal-like lineage as expected. Magnetic assembled 3D hDPSC cultures presented a consistent and controllable sphere-like morphology from 48h until 11 culture days and cell viability is improved with M3DB after day 3 (Fig. 2a, S2c, S2d and S2e). Also, these assembled 3D cultures expressed typical hDPSC and oral primordium genetic and protein markers throughout culture (Fig. 2b and c, respectively) including Nanog, CD90, CD29, Pitx1, KRT5, KRT14 and KRT19. Epithelial *adherens* junctions (E-cad and EpCAM) were also present as well as pan-neuronal markers (Fig. 2c), which promoted cell-to-cell interactions and viability of the 3D hDPSC construct.

### 3.2 M3DB supported the biofabrication of functional SG epithelial organoids

**Fig. 3:**
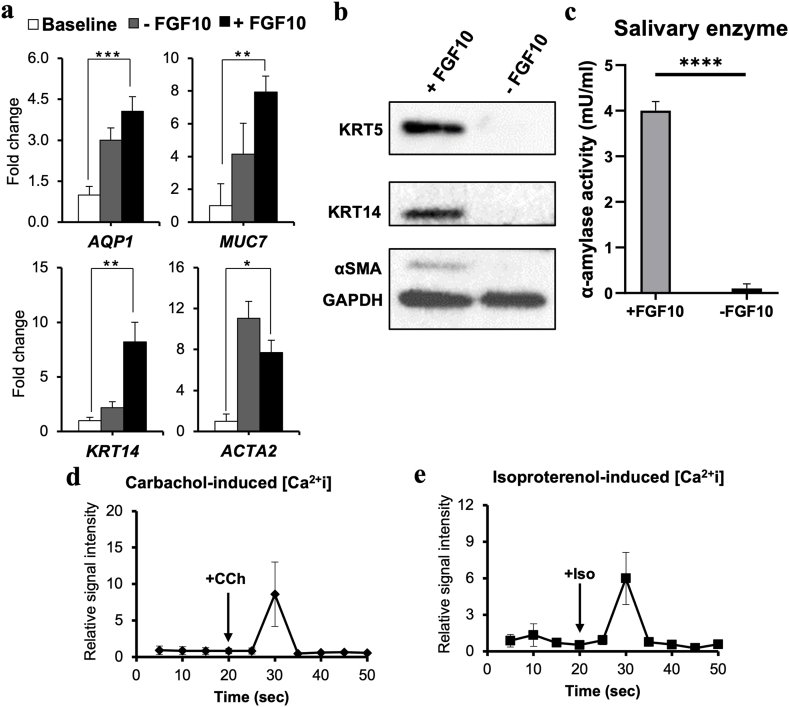
Phenotypic and functional characterization of 3D bio-assembled SG organoids. (**a**) SG assembled organoids formed by M3DB were enriched in SG-specific acinar epithelial, ductal and myoepithelial markers as per transcriptome analysis. Y-axis represent fold change in gene expression relative to baseline (24h of culture) and after normalizing to house-keeping gene (*Rps29).* Error bars are ±SEM from n = 4. *p < 0.05, **p < 0.01, ***p < 0.001 from one-way ANOVA comparisons with *Tukey* post-hoc. (**b**) Western blot assay of SG organoids induced by FGF10 (+FGF10) expressed SG epithelial and myoepithelial markers. “-FGF10” are undifferentiated M3DB cultures not supplemented with any SG key growth factors. (**c**) Expression of salivary α-amylase activity on the organoids after stimulation with FGF10. ***p < 0.0001 from *Student t*-test comparison from n = 4 samples. (**d-e**) Intracellular calcium influx in SG organoids (+FGF10) during (**d**) stimulation with Carbachol (CCh) 100 μM and (**e**) stimulation with Isoproterenol (Iso) 100 μM. Data are representative of 3–4 biological organoids and are presented as a relative signal intensity where each reading was normalized to baseline calcium influx (at unstimulated state). Please see supplementary information for more phenotypic and genotypic data.

Next, we hypothesized that M3DB would support the biofabrication of functional SG epithelial organoids. Hence, in the next set of experiments, organoids were assembled via M3DB and the diameter size of such was below 0.4 mm after 72 h of incubation (Fig. S2d), and remained constant during the whole culture duration time with no major differences between the ones with and without FGF10 supplementation (Fig. S2d). SG acinar epithelial markers (AQP1, MUC7, AMY1) and ductal epithelial and myoepithelial cell types (KRT5, KRT14, KRT19, αSMA/ACTA2), neuronal (CHRM3, TUBB3, NPY1R, NRTN) were present in the final organoids induced by FGF10 as confirmed by gene expression arrays (Fig. 3a, Fig. S4), Western blot (Fig. 3b) and in our previously reported immunofluorescent assays [16]. On the contrary, cultures without FGF10 supplementation did not express such SG-specific markers but undifferentiated markers instead (Figs. S3 and S4). Furthermore, the final SG organoids cultures produced enhanced α-amylase activity upon differentiation with FGF10 supplementation (Fig. 3c) as expected based on our earlier study [16]. The epithelial secretory function was confirmed via cholinergic stimulation with 100 μM Carbachol and via adrenergic stimulation with 100 μM Isoproterenol (Fig. 3d and e, respectively). Time-lapsed images showed the labeled calcium ion being mobilized in real time cultures of SG organoids (Fig. S5).

### 3.3 Exosomes were identified from EV isolated from hDPSC and SG organoids assembled via M3DB

**Fig. 4:**
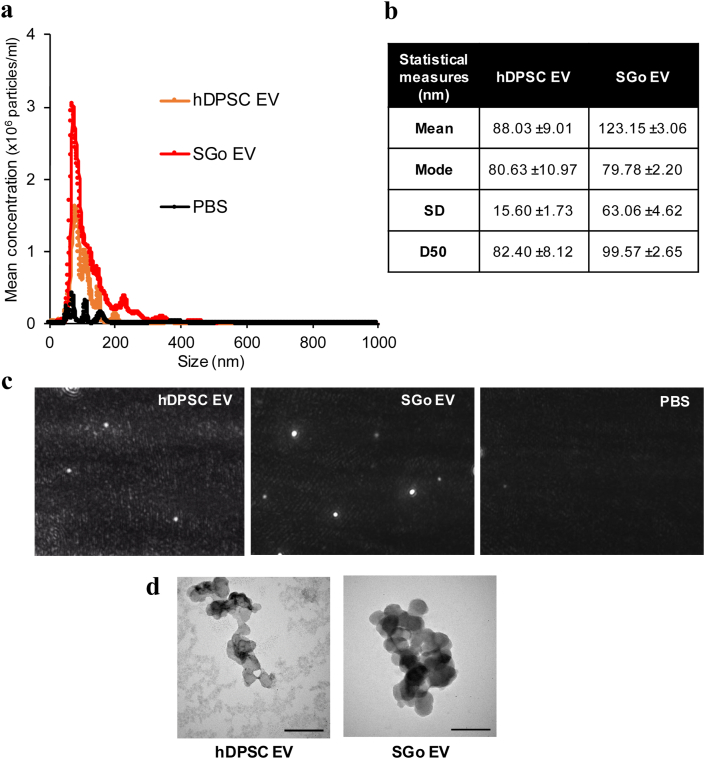
Size distribution, concentration and morphology of extracellular vesicles (exosomes) collected from the conditioned media of hDPSC and SGo cultures in M3DB platforms. (a–c) The size of hDPSC and SGo extracellular vesicles (EV) via nanoparticle tracking analysis (NTA) are within the exosome reported range (40–150 nm): (**a**) mean size distribution profile of hDPSC and SGo extracellular vesicles versus control buffer (PBS); (**b**) summary of particle size statistical measurements for the SGo secretome. The SD is a measure of the width (spread) of the size distribution profile. D50 value indicates the largest size in 50% of the particles. Data are presented as means ± SEM (*n* = 3), each sample was the representative average of 5 replicates; (**c**) Snapshot images of EV particles from hDPSC, SGo and PBS. (**d**) EV morphology by transmission electron microscopy. Scale bar: 100 nm.

Next, to better understand the paracrine factors released by magnetic bio-assembled hDPSC, we isolated and identified the EV from their conditioned media. The presence and characterization of EV was conducted after classical sequential centrifugation of the conditioned media (CM) derived from hDPSC and SG organoids (SGo). EV were within the typical exosome range for both hDPSC (mean diameter: 88.03 nm ± SD 15.60 nm) and SGo (mean diameter 123.15 nm ± SD 63.06 nm) (Fig. 4a and 4b) as per NTA. The majority of hDPSC EV were slightly smaller than SGo EV, which could also be depicted in the statistical mode and D50 values (Fig. 4b), in video snapshots of Brownian motion particles (Fig. 4c) and in TEM micrographs (Fig. 4d). The presence of exosomes and their round morphology was confirmed by TEM, which showed micrographs with diameter sizes varying between 50 and 150 nm (Fig. 4d). Both TEM and NTA confirmed that all EV were consistently within the standard exosome reported range (40–150 nm) [31,32]. Moreover, exosome-specific markers ALIX and TSG101 were present in hDPSC EV and SGo EV in immunoblots (Fig. S7).

### 3.4 Treatment with hDPSC-derived exosomes promoted marginal epithelial growth repair

**Fig. 5:**
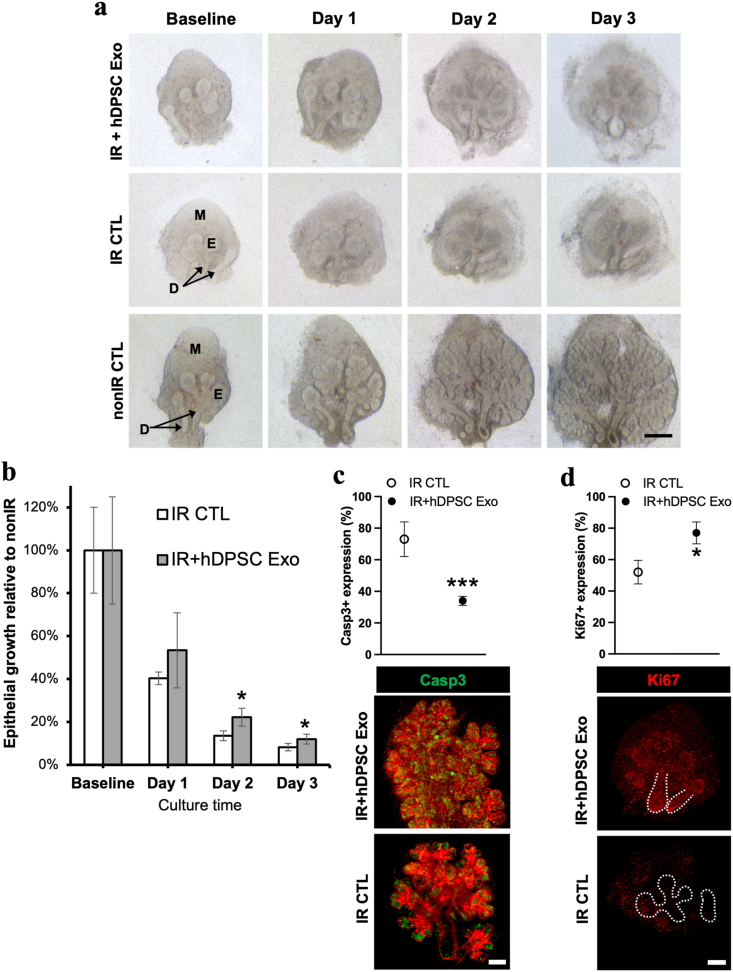
Epithelial growth is minimally rescued in irradiated SG after treatment with exosomes from hDPSC cultures (hDPSC Exo) in M3DB platforms. (**a**) Representative bright field images acquired from each SG pair after hDPSC Exo treatment is shown, as well as controls (IR: irradiated; nonIR: non-irradiated). Scale bar: 200 μm. M: mesenchyme. E: Epithelial bud. D: Ducts. A: Apoptosis. (**b**) SG growth after hDPSC Exo treatment upon radiation damage (IR). The final epithelial growth percentage was calculated by determining the Spooner's ratio of that experimental group (epithelial buds at time point/buds at baseline) and dividing it to the Spooner's ratio of nonIR group. Data represent mean ± SEM from *n* = 4–5. *p < 0.05 when compared to IR CTL group using paired Student's *t-*test. (**c-d**) Quantification of apoptotic and mitotic cells by immunofluorescence and ImageJ. The expression of cleaved Caspase 3 (Casp3) (**c**) and Ki67 (**d**) were normalized to total nuclei. (**c**) IHC representative images showing Cas3+ cells (green) inside the buds surrounded by perlecan (red) basement membrane. Scale bar: 100 μm. (**c**) IHC representative images showing Ki67+ cells (green). Merged images with nuclei and other markers are displayed in Supplementary Fig. S7. Scale bar: 100 μm. Data represent mean ± SEM from *n* = 4–5. *p < 0.05 and ***p < 0.001 when compared to IR CTL group using unpaired *t*-test.

To determine the epithelial repair potential of hDPSC-derived exosomes, *ex vivo* fetal SG models were utilized, and such gland explants were exposed to radiation to produce epithelial damage. Exosomes and other particles (adenoviral-based) in these *ex vivo* epithelial growth repair models, can travel from the media (below the glands) through 200 nm pores in Whatman membranes and reach the injured glands on the opposite side of the membrane [33]. Treatment of irradiated SG epithelia with hDPSC-derived exosomes (hDPSC Exo) showed a marginal rescue of epithelial growth when compared to IR controls (∼4–5% after 3 days). This could be related with the presence of apoptotic cells (cleaved Caspase 3+ cells in Fig. 5c). Despite such findings, mitotic cells (Ki67^+^) significantly increased with hDPSC Exo (Fig. 5d) and there was a slight increase in the stem/progenitor cell population (SOX2^+^) and the neuronal network (β3-tubulin^+^) (Fig. S8).

### 3.5 Administration of SGo-derived exosomes stimulated epithelial growth and innervation

**Fig. 6:**
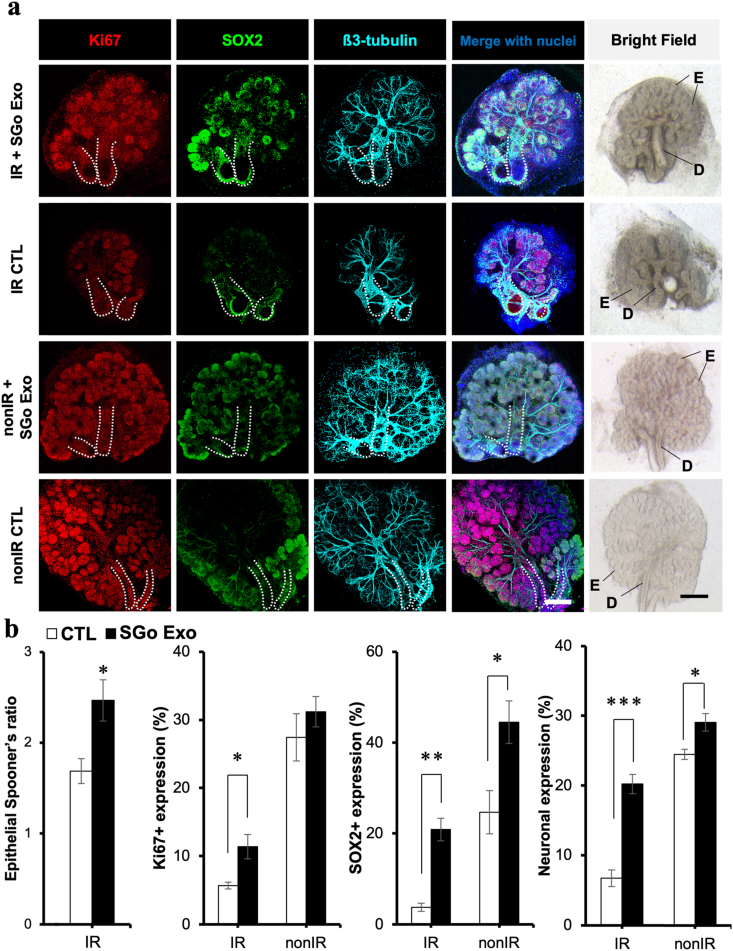
Epithelial growth, mitosis, epithelial progenitors and innervation was increased after treatment with exosomes from SG organoids in radiation-induced SG injury models. (**a**) Bright field and immunofluorescent images of the SG from IR and nonIR models after SG organoid exosome (SGo Exo) treatment. Glands were immunostained and fluorescently labeled for mitotic cells (Ki67), SG progenitors (SOX2), neurons (β3-tubulin) and counterstained with a nuclear dye (Hoechst 33342). Scale bars: 200 μm. E: Epithelial acinar buds, D: Ducts, SGo: salivary gland organoid, IR: irradiated; nonIR: non-irradiated. (**b**) Quantification of epithelial bud Spooner's ratio, mitotic cells, SG progenitors and neuronal expression by whole-mount immunohistochemistry and ImageJ or Imaris software in 5 random regions of interest. Data were normalized to nuclear content or explant are and represent mean ± SEM from *n* = 5–6. Unpaired t-tests with Welch's correction were performed: *p < 0.05, **p < 0.01, ****p < 0.0001.

Next, SGo-derived exosomes were added to the SG damaged explants to assess epithelial and neuronal repair. Treatment with SGo-derived exosomes extensively rescued epithelial growth in irradiated SG epithelial models (Fig. 6a), and significantly increased the number of mitotic cells, SG progenitors and the neuronal network (Fig. 6b). In normal non-irradiated (nonIR) SG epithelial models, the number of SG progenitors and the neuronal network area were enhanced significantly with such exosomes, but not the number of mitotic cells (Fig. 6a and b). These outcomes in IR and nonIR SG models indicate beneficial epithelial growth effects promoted by SGo exosomes.

**Fig. 7:**
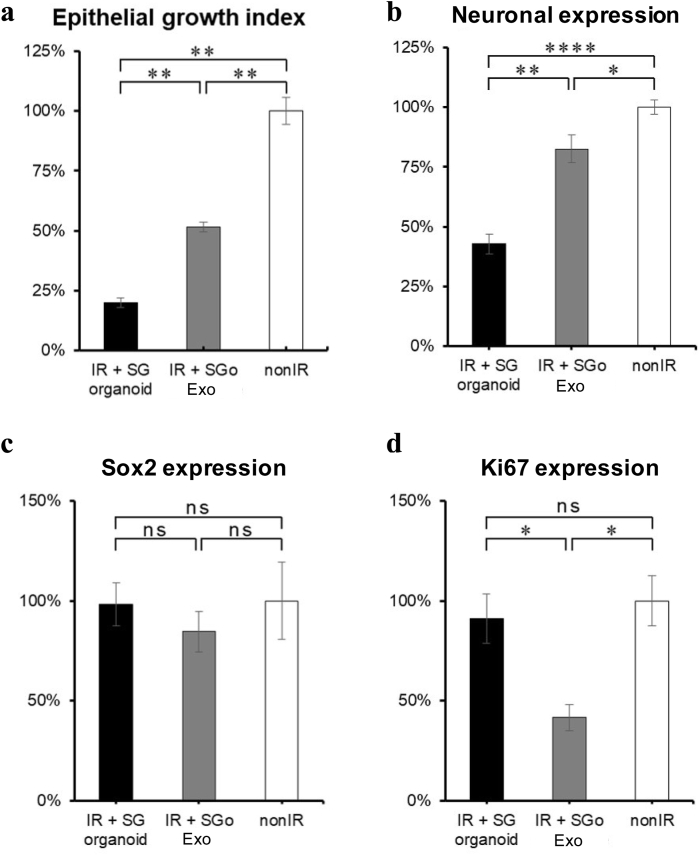
SGo-derived exosomes promoted greater epithelial and neuronal rescue when compared to control SG organoids. (**a**) Epithelial growth was calculated by determining the Spooner's epithelial growth ratio that experimental group (epithelial buds at time point/buds at baseline) and dividing it to the Spooner's ratio of nonIR group. (b–d) Quantification of cells expressing specific SG protein markers at different treatment groups by whole-mount immunohistochemistry and ImageJ software: (**b**) β3-tubulin + neuronal expression, (**c**) SOX2 expression, (**d**) Ki67 expression. Y-axis display values that are normalized to the total nuclear area (Hoechst 33342 stained cells) of the explants for both fetal glands (submandibular and sublingual). The integrated density of the fluorescence signal was normalized to the number of Hoechst 33342 stained cells using ImageJ software from (**b**) through (**d**). Data are means ± SEM (*n* = 5–6). Unpaired t-tests were performed: ns: no statistical difference, *p < 0.05, **p < 0.01, ****p < 0.0001. SGo: salivary gland organoid. IR: irradiated. nonIR: non-irradiated.

Moreover, when comparing with control transplanted SG organoids, treatment with SGo-derived exosomes was found to further promote epithelial bud growth in irradiated SG epithelia and enhance the epithelial rescue from 25% (in SG organoids) up to approximately 60% (Fig. 7a). This was perhaps related to an enlarged parasympathetic neuronal network in the treatment group with SGo-derived exosomes (Fig. 7b, S8, and S9b), since there was no difference in the stem/progenitor cell SOX2^+^ marker (Fig. 7c) between all treatment and non-irradiated groups. SOX2 expression does become restricted to the ducts and the sublingual gland with the progression of culture time as expected [26]. The parasympathetic neuronal network can maintain the SG epithelial progenitors and rescue the epithelial growth after radiation injury as per previous reports [24,25]. Despite these findings, the number of mitotic cells decreased with SGo exosome treatment (Ki67^+^ cells, Fig. 7d).

### 3.6 Identification of exosomal cargo proteins

**Fig. 8:**
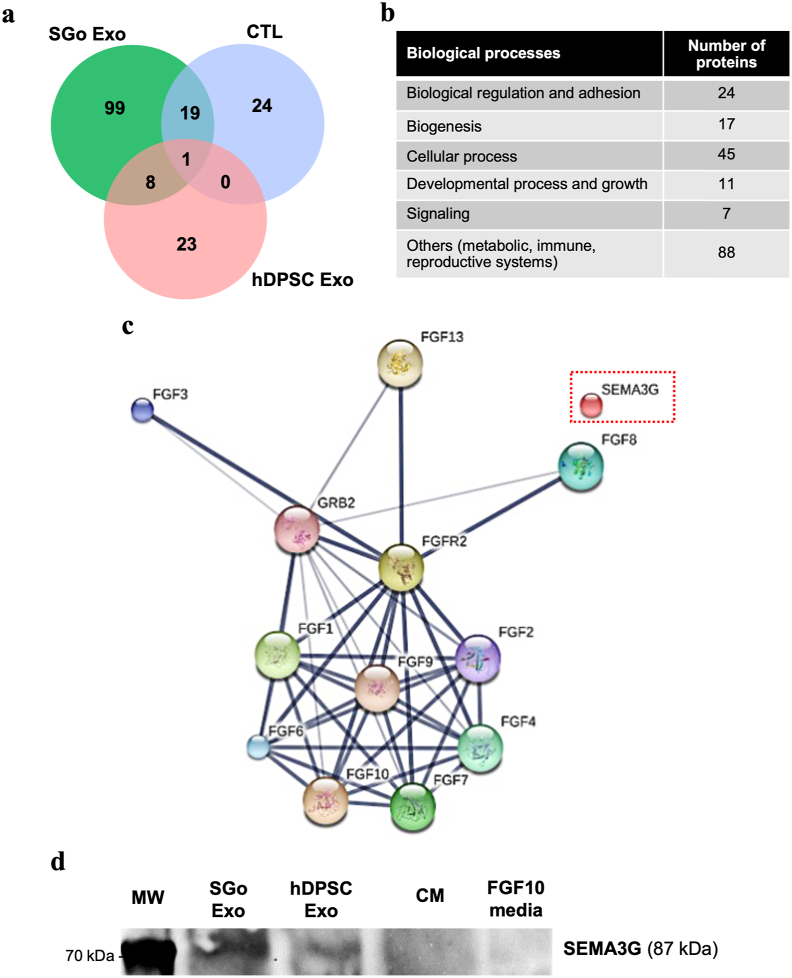
Differentially expressed exosomal cargo proteins derived from SG organoids are both known and undocumented FGF10 downstream molecular targets. **(a)** Venn diagram from the LC-MS/MS analysis displaying the differentially expressed exosomal/EV cargo proteins that are unique to exosomes derived from SG organoids (99 proteins in “SGo Exo” group which differentiation was driven by FGF10) or shared by the two exosomal treatments (SGo and hDPSC) and a conditioned media control where hDPSC were maintained in epithelial differentiation media without FGF10 (CTL). **(b)** Biological processes induced by the 99 exosomal proteins that are differentially expressed in SG organoids assembled by M3DB. These were extracted by the Panther classification system. **(c)** Biological interactions of the differentially expressed exosomal proteins with FGF10 and other downstream molecular pathways analyzed by the Stitch software that are relevant to SG development. Semaphorins like SEMA3G (dashed red box frame) were also identified as novel and less established FGF10 targets. **(d)** Western blot assay confirming the differentially expressed SEMA3G in the exosome protein cargo from SG organoids. MW: molecular weight standard (70 kDa). CM: conditioned media without EV (negative control). FG10 media: fresh epithelial differentiation media with FGF10 (400 ng/ml).

**Fig. 9:**
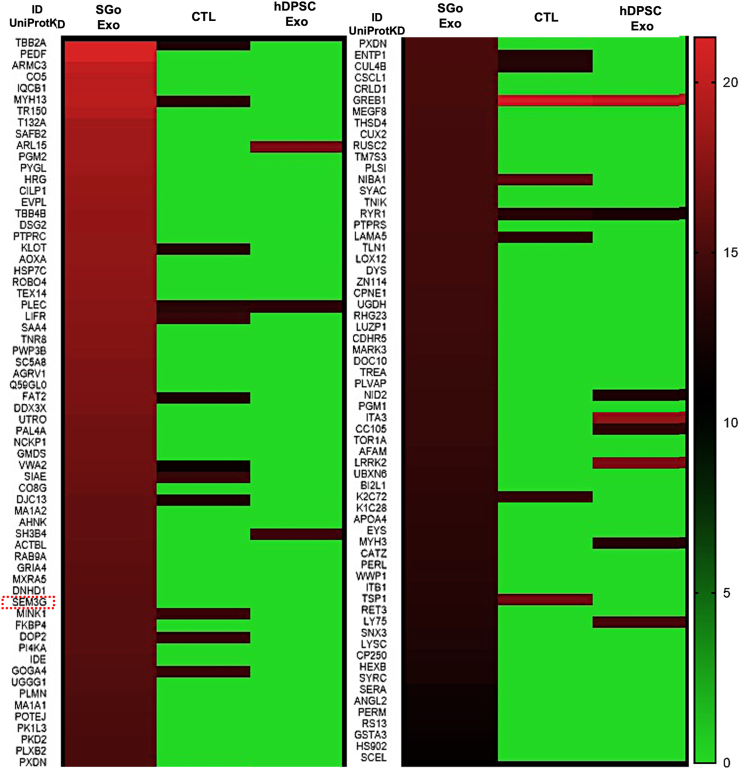
List of differentially expressed exosomal cargo proteins derived from SG organoids. Heat map displaying the mean fold change of the 99 exosome proteins normalized to each fresh reference culture media (n = 3). All 3 samples per group were averaged and displayed on the heatmap. The universal protein knowledge database (UniProtKD) was utilized. Semaphorins like SEMA3G (dashed red box frame) were also identified as novel and less established FGF10 target and all such markers significantly upregulated (*p < 0.05).

To identify the paracrine factors in the EV cargo after FGF10 stimulation, comprehensive proteome assessments were made. The mass spectrometry proteome-based analysis showed that 99 cargo proteins specific to exosomes and/or EV were either upregulated or downregulated in the exosomes derived from SG organoids (“SGo Exo”), which did not overlap with exosomal proteins from other culture conditions (“CTL” and “hDPSC Exo”) (Fig. 8a). These differentially expressed exosome proteins were analyzed by Panther software and were found involved in many cellular pathways such as biological regulation and adhesion, biogenesis, development, and cellular growth, and signaling downstream of FGF10 (Fig. 8b). Stitch analysis of SGo-derived exosomes displayed several well-established relationships and interactions with FGF10 molecular targets involved in cell growth and development, as well as poorly recognized molecular targets like semaphorins (SEM3G or SEMA3G alias) (Fig. 8c). Specific protein targets were predicted as functional proteins and were validated by Western blot (Fig. 8d). The fold change of these 99 differentially expressed exosomal proteins is displayed on a heat map (Fig. 9).

## 4 Discussion

Magnetic bioassembly platforms can contribute to the biofabrication and up-scale production of SG human and porcine organoids as previously reported by our group [16,17]; but the functional role of EV secreted from these bioassembly platforms remains to be evaluated in SG epithelial repair [[41], [42], [43]]. In this study, exosomes derived from human SG assembled organoids (SGo) had a more prominent paracrine role in epithelial SG growth and repair (up to 60%) when compared to exosomes developed from hDPSC 3D M3DB cultures (up to 15%) and SG organoid transplants (up to 25%). Human ASC from dental pulp (hDPSC) or bone marrow are known to generate bioactive EV and/or intracellular molecules that can repair many tissues or organs including SG [27,28,43,44]. Although, EV developed from hDPSC's secretome in conditioned media were never evaluated in bioassembly platforms and SG organoid cultures. Thus, this is the first report addressing this knowledge gap.

Our recently developed magnetic bioassembly (M3DB) platforms allowed for a robust culture of hDPSC tagged with magnetic nanoparticles to fabricate hDPSC 3D cultures and functional SG-like epithelial organoids (Fig. 2, Fig. 3, and S2-S5). Moreover, the particular MNP used in our bioassembly process were nontoxic as compared to commercially used bioinks [18,[45], [46], [47], [48]], and did not exhibit any meaningful impact on cell proliferation. In addition, these MNP did not induce any inflammatory response in previous works [17,49,50]. The cellular phenotype and secretory function of FGF10-stimulated organoids showed how important is the role of FGF10 on SG morphogenesis and innervation as previously established [16,20,51,52]. The presence of a diverse number of SG epithelial markers in the organoids was confirmed by transcriptional and proteomic profiling, including the acinar secretory units (Fig. 3a–b, and S2d, S3-S4). Furthermore, these SGo were able to produce the salivary enzyme α-amylase and respond to different autonomic neurotransmitters (Fig. 3c–e and S5). Extraction and analysis of EV from hDPSC and SG organoids identified EV at the exosome range (Fig. 4a–d). The morphology was cup-shaped and within the small EV size (<127 nm) as categorized by the ISEV [32,53].

In this study, the treatment with hDPSC-derived exosomes had a minimal effect on epithelial growth, whereas SGo-derived exosomes rescued up to 60% of growth (Fig. 7a) and significantly contributed to the epithelial repair by promoting mitosis, the enrichment of epithelial stem/progenitor cell niches and an enlargement of the neuronal network (Fig. 6b). The functional role of hDPSC EV has been reported particularly in the dental literature as they can activate the regeneration of dental pulp tissue and induce stem cell differentiation [43]. Recently, EV studies on the *ex vivo* fetal SG organ highlight the role of exosomal miRNAs during their transportation from the mesenchyme to epithelium in the gland organogenesis process [31,54]. These fetal or germ glands have been utilized by many researchers to understand what cellular niches contribute to the epithelial repair process in irradiation models, since radiation injury can have a detrimental negative impact on epithelial growth, development and maturation [15,[24], [25], [26],^33^]. Adult glands may constitute more appropriate models for clinical translation; however, many adult mouse strains appear to display distinctive levels of epithelial injury after radiation therapy [55]. This fact is probably due to diverse postnatal epithelial sensitivities to radiation. Also, in our fetal SG cultures, the mesenchyme was not removed since it would damage or completely detach the neuronal network from the epithelia. Interestingly, the number of mitotic cells decreased with SGo-derived exosome treatment in comparison with transplanted SG organoids, though such finding is potentially related to a faster differentiation or maturation of epithelial progenitors in the presence of FGF10 downstream molecular targets as depicted by mass spectrometry of the exosomal proteins (Fig. 8), which may consequently lead to a loss of the progenitors’ self-renewal ability.

In addition, several molecules in the exosome/EV cargo protein family were upregulated in the proteomic analysis and were forecasted as a functional proteins with important roles in cell growth and development, particularly those with strong interactions and relationships with the FGF10 protein family. Interestingly, one class 3 semaphorin protein (SEMA3G) was identified by LC-MS/MS and immunoblots as a novel functional protein with less known interactions with FGF10 downstream molecular targets. Class 3 semaphorins (SEMA3) are a family of membrane‐bound proteins initially recognized as important factors for successful axonal and neuronal growth [56]. In the normal SG, SEMA3 proteins are highly expressed in the acinar compartment of serous glands but are rarely expressed in the ductal network [56]. During SG development, cleft formation is accelerated by SEMA3 signaling pathways in epithelial cells [57], although future studies are necessary to investigate the exact role of SEMA3 proteins in SG epithelial repair and regeneration strategies. Though, one could speculate that the amelioration of the epithelial damage maybe occurring via the protein cargo molecules of the SGo exosome/EV that were found differentially expressed relative to their hDPSC exosome/EV counterparts (which did not provide a significant damage rescue).

The evidence presented in this study suggests that exosomes derived from SG organoids assembled by M3DB can be a viable strategy for the amelioration of SG epithelial damage; however, their precise role in *in* vivo SG regenerative models with different radiotherapy modalities commonly utilized for late-stage head and neck cancers remains to be tested. In addition, exosomes and EV are complex biological cues/systems comprising a great number of molecules in their cargo that may raise multiple safety and predictability issues. Addressing these concerns in our future research will help to further define the unique functional roles of exosome cargo proteins identified in this study (i.e. FGF10 downstream targets and semaphorins), and perhaps incorporate these functional cargo proteins into organoids or transplants to synergistically drive SG epithelial repair.

## CRediT authorship contribution statement

**Ajjima Chansaenroj:** Methodology, Investigation, Formal analysis, Validation, Data curation, Writing – original draft, Writing – review & editing, Visualization. **Christabella Adine:** Conceptualization, Methodology, Investigation, Formal analysis, Validation, Writing – review & editing, Visualization. **Sawanya Charoenlappanit:** Methodology, Investigation, Resources, Writing – review & editing. **Sittiruk Roytrakul:** Investigation, Resources, Software, Validation. **Ladawan Sariya:** Investigation, Resources, Writing – review & editing. **Thanaphum Osathanon:** Resources, Writing – review & editing. **Sasitorn Rungarunlert:** Resources, Writing – review & editing, Funding acquisition. **Ganokon Urkasemsin:** Resources, Writing – review & editing, Supervision, Funding acquisition. **Risa Chaisuparat:** Resources, Writing – review & editing, Project administration, Funding acquisition. **Supansa Yodmuang:** Resources, Writing – review & editing, Project administration, Funding acquisition. **Glauco R. Souza:** Resources, Writing – review & editing, Supervision. **João Nuno Ferreira:** Conceptualization, Validation, Resources, Writing – review & editing, Visualization, Supervision, Project administration, Funding acquisition.

## Declaration of competing interest

The authors declare that they have no known competing financial interests or personal relationships that could have appeared to influence the work reported in this paper.
