# Supplementary material for: Magnetic bioassembly platforms towards the generation of extracellular vesicles from human salivary gland functional organoids for epithelial repair
Source: Bioact Mater. 2022 Feb 16;18:151–63. doi: 10.1016/j.bioactmat.2022.02.007 (PMC8961305; doi:10.1016/j.bioactmat.2022.02.007)
Supplement: Multimedia component 1 [file mmc1.pdf]

Supplementary data

Table S1.

	Antibody	Dilution	Manufacturer
**Conjugated Antibodies for FC**	Pacific Blue anti-CD73	1:40	Biolegend, USA
	FITC anti-CD105	1:80	Biolegend, USA
	Alexa Fluor 647 anti-CD34	1:300	Biolegend, USA
	Pacific Blue anti-CD45	1:200	Biolegend, USA
**Primary Antibodies for IHC**	anti-KRT5	1:200	Abcam, UK
	anti-KRT14	1:200	Abcam, UK
	anti-KRT19	1:50	Novus Biologicals, USA
	anti-E-cadherin	1:200	Cell signaling, USA
	Anti-EpCAM	1:50	Thermo Fisher, USA
	anti-Ki67	1:100	Thermo Fisher, USA
	anti-Sox2	1:100	Santa Cruz, USA
	anti-ß3 tubulin	1:200	R&D systems, USA
**Primary Antibodies for WB**	anti-KRT5	1:1,000	Abcam, UK
	anti-KRT14	1:2,000	Abcam, UK
	anti-αSMA	1:400	Abcam, UK
	anti-GAPDH	1:2,000	Sigma, USA
	anti-ALIX	1:500	Abcam, UK
	anti-TSG101	1:500	Abcam, UK
	anti-SEMA3G	1:250	Abcam, UK

Table S1. List of antibodies (conjugated and unconjugated) used for flow cytometry (FC), whole-mount immunohistochemistry (IHC) and western blot (WB).

Table S2.

Gene	Forward sequence	Reverse sequence
s29	CAATATGTGCCGCCAGTGT	GAAGGAAGAGCATTTAGTCCAACT
NANOG	TGATTTGTGGGCCTGAAGAAAA	GAGGCATCTCAGCAGAAGACA
SOX2	GCCGAGTGGAAACTTTTGTCG	GGCAGCGTGTACTTATCCTTCT
PAX9	GGTGAACGGGTTGGAGAAG	CTGTAGGTCATGTAAGGCGAC
DLX2	CTTACTCCGCCAAGAGCAG	TCCCGTTCACTATCCGAATTTC
PITX1	GTCTGACACGGAGCTGC	TGGCTTGTGAAGTGCGTAC
ASCL3	GGACAACAGAGGCAACTCTAGT	AGGGGCAGGGTTCACTGTAAT
KIT	GTCTCCACCATCCATCCA	TCCATTCATTCTGCTTATTCTCA
CD29	TGTAAGGAGAAGGATGTTGACG	CAACCACACCAGCTACAATTG
KRT5	CGTGCCGCAGTTCTATATTCT	ACTTTGGGTTCTCGTGTCAG
KRT14	CACAGATCCCACTGGAAGAT	GATAATGAAGCTGTATTGATTGCC
AMY1	CTCGGCACAGTTATTCGCAAGTGG	ACAGCCTAGCATCCCAGAAGGT
MUC7	GCTCCACCAGAGACCACAG	GCTGAGGCGGATGATGGG
FGFR2B	AAGTGCTGGCTCTGTTCAATGT	GCCTGCCCTATATAATTGGAGACC
AQP1	CTGGGCATCGAGATCATCGG	ATCCCACAGCCAGTGTAGTCA
KRT19	CTGCCTCCAAGGTCCTCT	CCCATCCCTCTACCCAGAAG
ACTA2	GACTTCCGCTTCAATTCC	GTTAGGACCTTCCCTCAG
CD90	GAGATCCCAGAACCATGAACC	TGCTGGTATTCTCATGGCG
NES	GGTCTCTTTTCTCTTCCGTCC	CTCCCACATCTGAAACGACTC
CDH1	CCAAAGACAGAGCGGAACTATG	CCTCAATCATCCTCAGCATCAG
PECAM	TGAGACGGAGATTCGCTCTTGTTG	GCTGAGACAGGAGAACTGCTTGAA
Tubb3	CGAAGCCAGCAGTGTCTAAA	GGAGGACGAGGCCATAAATA
CHRM3	CATCATGAATCGATGGGCCT	CATCATGAATCGATGGGCCT
NPY1R	GAGGCGATGTGTAAGTTGAATCC	TGGAACGGCTCATCAGTCATT
NRTN	CCCTGCCTGTGATGCCATTCTC	GAGCCGATGACAAGGTCCAGACT

Table S2. Oligonucleotide forward and reverse primer sequences optimized for mRNA in human SG tissues and used for qPCR.

Figure S1.

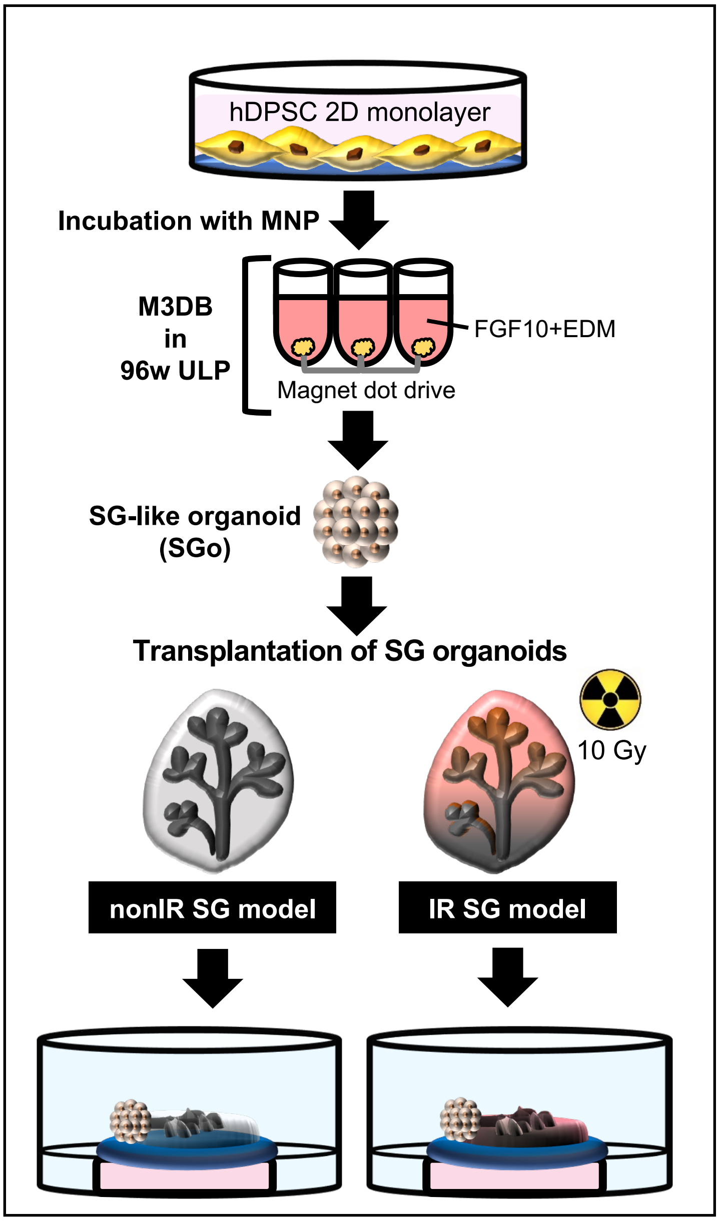

Figure S1. Magnetic three-dimensional bioassembly (M3DB) strategy to generate the SG organoids as controls to compare with exosomal treatments. hDPSC were cultured as a monolayer overnight and tagged with magnetic nanoparticles (MNP). Next, magnetized cells were dissociated into single cell solutions, and assembled with a magnetic drive with 96 neodymium dot magnets (0.062500 OD) for a 96-well ultra-low attachment plate (96w ULP). hDPSC were maintained at the 96w ULP at the bottom of each well and exposed to a field strength of 120 G (10 pN), which kept them at ~4 mm from the magnet. Transplantation of SG organoid into non-irradiated SG (nonIR) and irradiated (IR) SG organ models. SG: salivary glands; SGo: salivary gland-like organoid; IR: irradiated. nonIR: non-irradiated. EDM: epithelial differentiation media. FGF10: Fibroblast growth factor 10.

Figure S2.

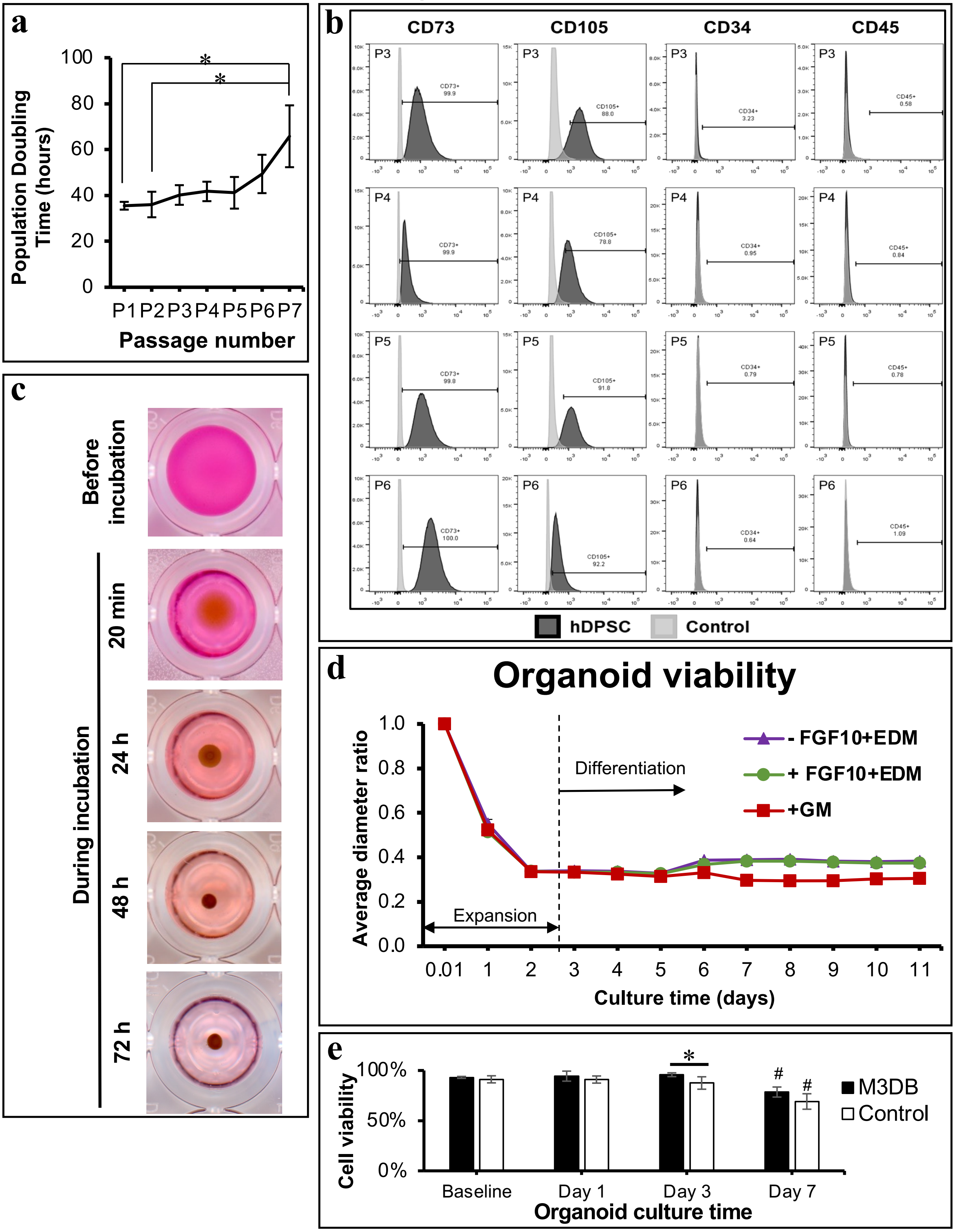

Figure S2. MSC and non-MSC surface markers and viability of hDPSC and organoids before and after M3DB. (a) Quantification of hDPSC population doubling time (PDT). Data are presented as means ± SEM (n = 3-6). One-way analysis of variance (ANOVA) with post-hoc Dunnett's: *$p < 0.05$ when compared between passages. (b) Representative flow cytometry histograms overlays showing MSC and hematopoietic cell surface marker expression in hDPSC at different passages. Controls immunostained with respective isotype IgG. (c) High-throughput workflow and organoid viability screening using high resolution scanner before and during hDPSC incubation in M3DB platform. (d) Quantification of organoid viability by assessing diameter during the entire culture (11 days) with and without FGF10 supplementation in epithelial differentiation media (EDM) as compared with hDPSC in growth media (GM). n = 8. Data represent mean ±SD. Diameter is expected to decrease with increased cell viability due to strong cell-cell epithelial interactions. (e) Cell viability in the organoids measured by Ki67 IHC relative to total nuclei. N = 4-5. Data are means ±SD. Student's t-test with Welch's correction: *$p<0.05$ when comparing M3DB versus Control. #$p<0.05$ when comparing between timepoints.

Figure S3.

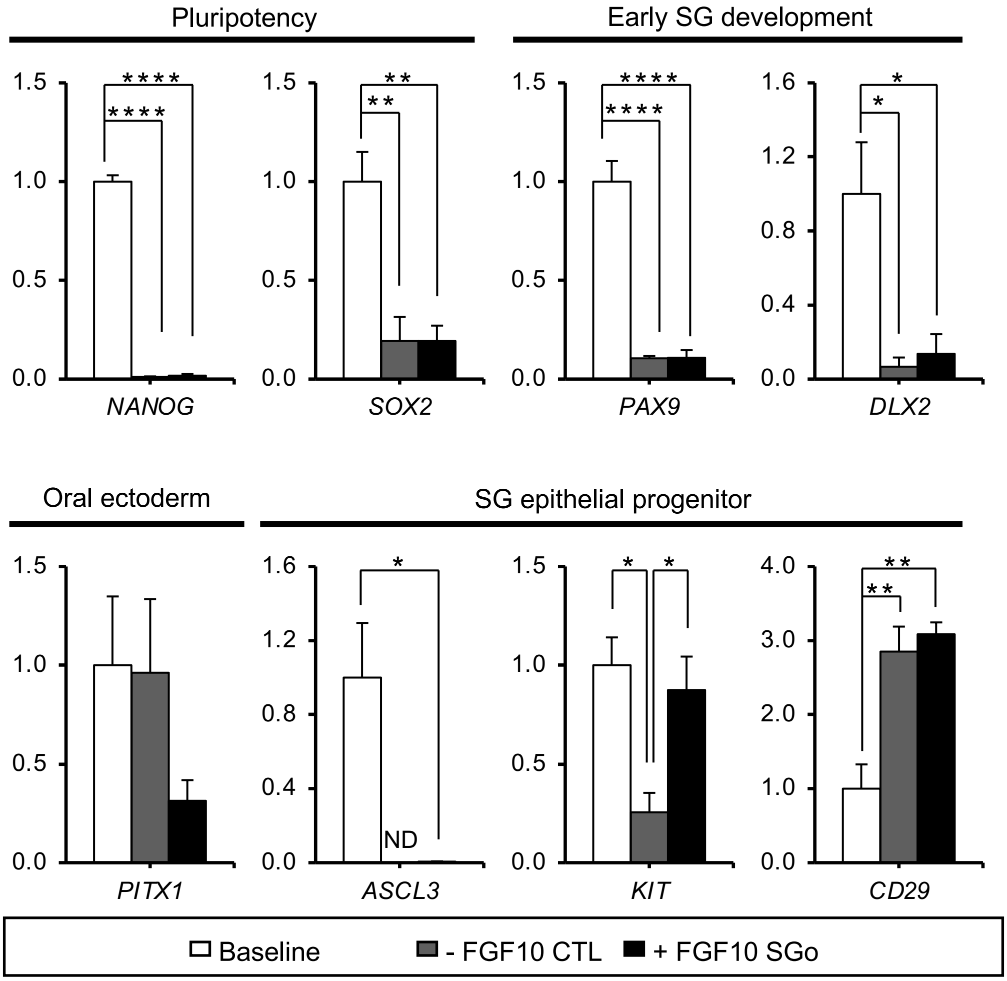

Figure S3. Mature SG organoids after differentiation presented a downregulation of pluripotency genetic markers and the markers involved in early SG development. Transcriptome analysis of spheroids after SG differentiation stage. Fold change values are normalized to the housekeeping gene *s29* and compared to undifferentiated spheroids at baseline. Spheroid groups are differentiated with epithelial differentiation media for 8 days without and with FGF10 (400 ng/ml). Data are presented as means ± SEM (n = 3-8). One-way analysis of variance (ANOVA) with post-hoc Tukey test were performed: *p < 0.05, ** p <0.01, ****p < 0.0001 when compared between groups. FGF10: fibroblast growth factor-10. EDM: epithelial differentiation media. ND: not detected.

Figure S4.

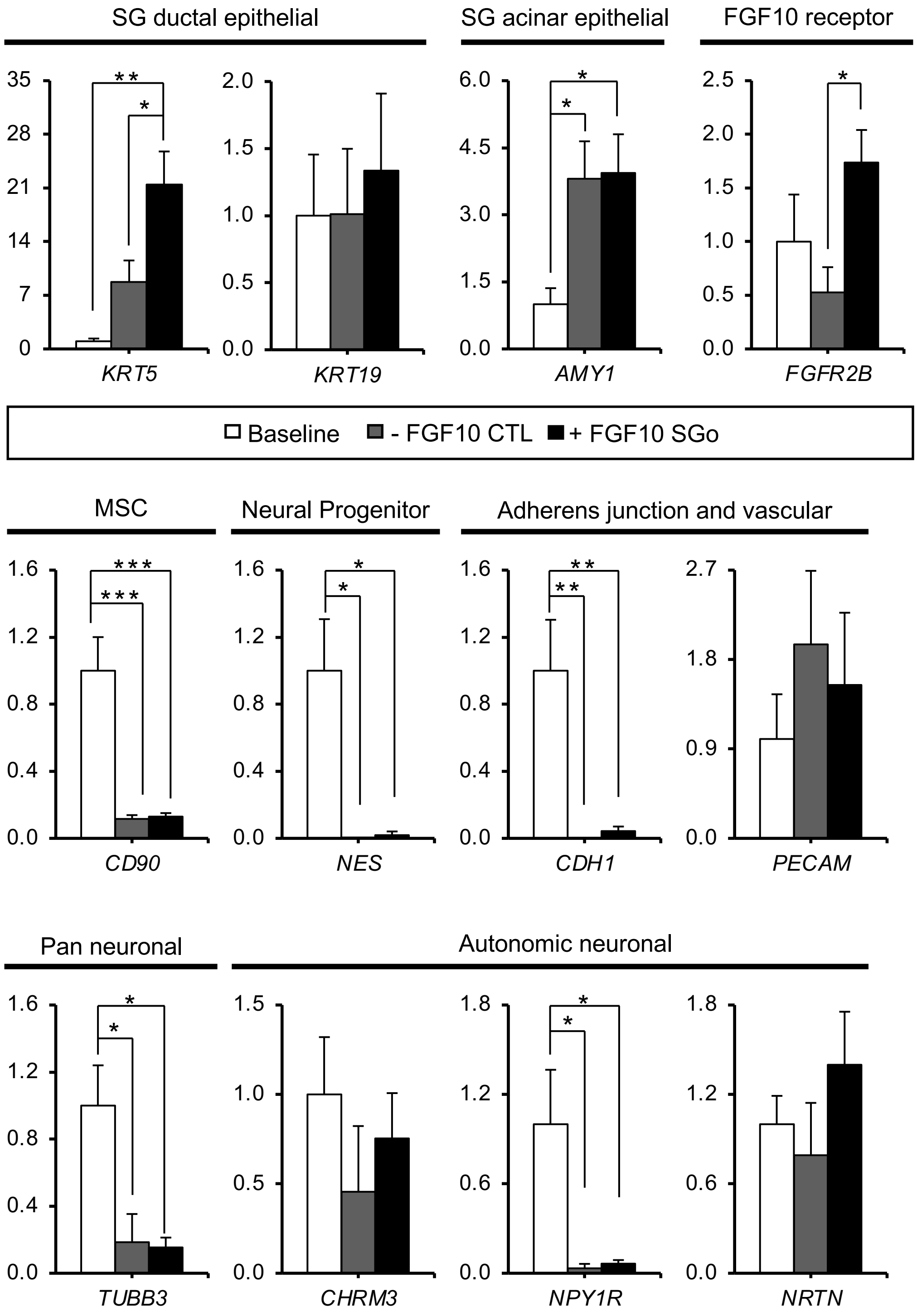

Figure S4. Organoids presented an upregulation of different SG epithelial genetic markers (acinar, ductal) after SG epithelial differentiation and expressed neuronal, epithelial *adherens* junctions, and vascular markers. Transcriptome analysis of spheroids after SG differentiation stage. Fold change values are normalized to the housekeeping gene *s29* and compared to undifferentiated spheroids at baseline. Spheroid groups are differentiated for 8 days without and with FGF10 (400 ng/ml). Data are presented as means ± SEM (n = 3-8). One-way analysis of variance (ANOVA) with post-hoc Tukey test were performed: *$p < 0.05$, **$p < 0.01$, ***$p < 0.001$ when compared between groups. FGF10: fibroblast growth factor-10.

FIGURE S5

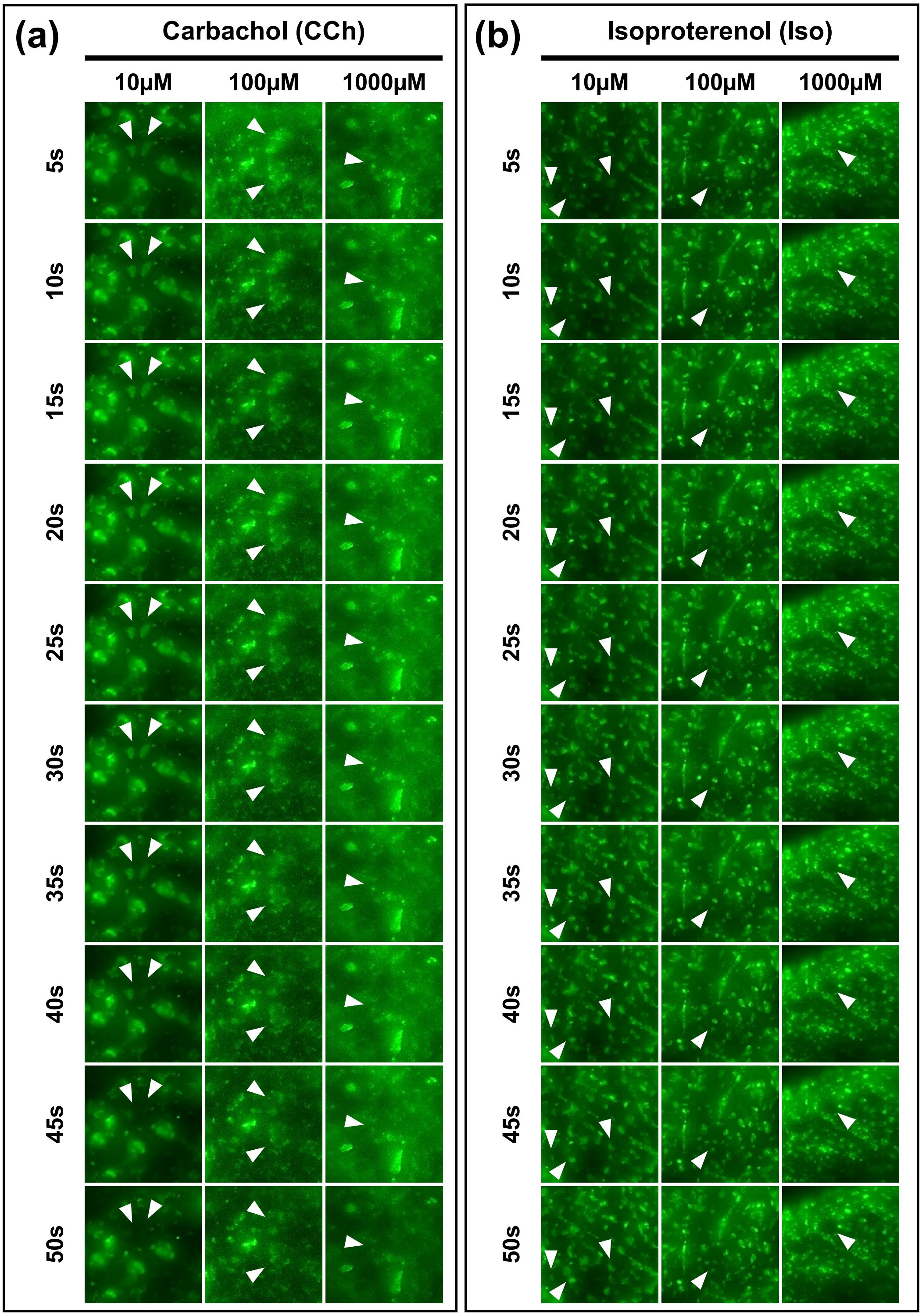

Figure S5. Representative microscopy time-lapse images showing calcium ion [Ca^{2+}_i] mobilization in real time cultures of SG organoids exposed to FGF10 after calcium fluorescence labeling (in green) before and after adrenergic stimulation with (a) muscarinic agonist, Carbachol (CCh) (10–1000 µM) (b) adrenergic agonist Isoproterenol (Iso) (10–1000 µM). Magnification: 10x.

Figure S6.

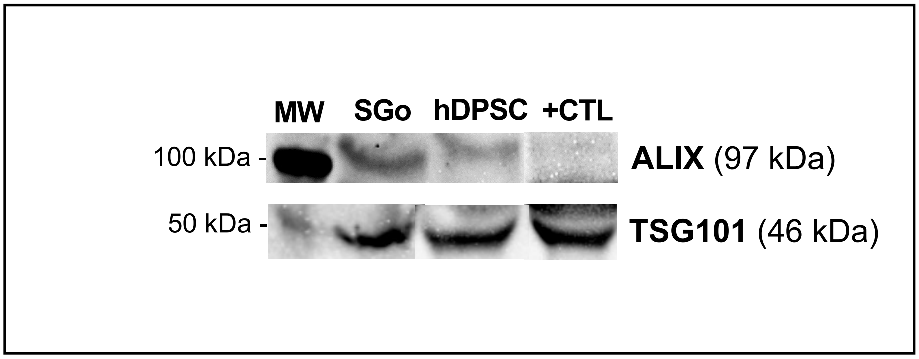

Figure S6. Western blot assay of exosome markers ALIX and TSG101 in SG M3DB organoids (SGo), hDPSC M3DB cultures (hDPSC). Positive controls (+CTL) are exosomes from the MCF7 epithelial breast cancer cell line. MW: molecular weight standards (100 kDa and 50 kDa).

Figure S7

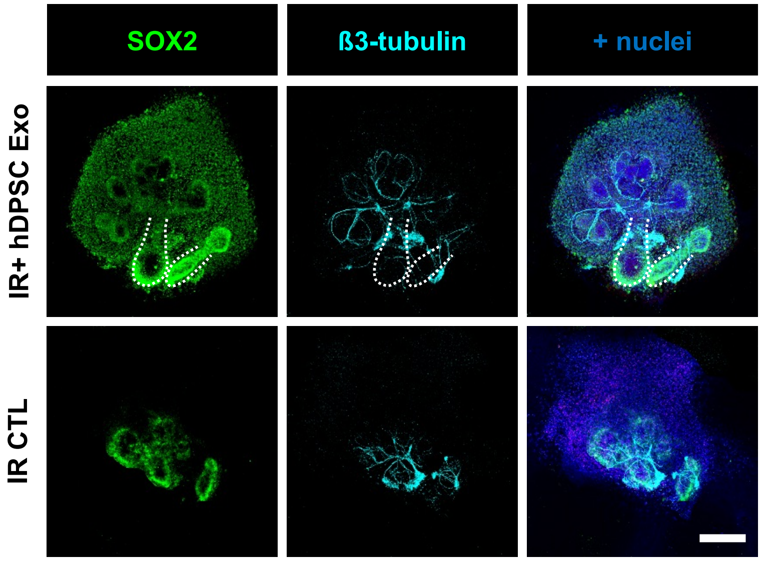

Figure S7. Progenitor cells and neuronal network were marginally rescued in irradiated SG after treatment with exosomes from hDPSC M3DB cultures. Representative maximum intensity images of mouse SG of each group immunostained with SG progenitors (SOX2), neurons (ß3-tubulin), and counterstained with a nuclear dye. Dotted white line denotes the SG ducts. Scale bar: 200 µm. IR: irradiation. CTL: control.

Figure S8.

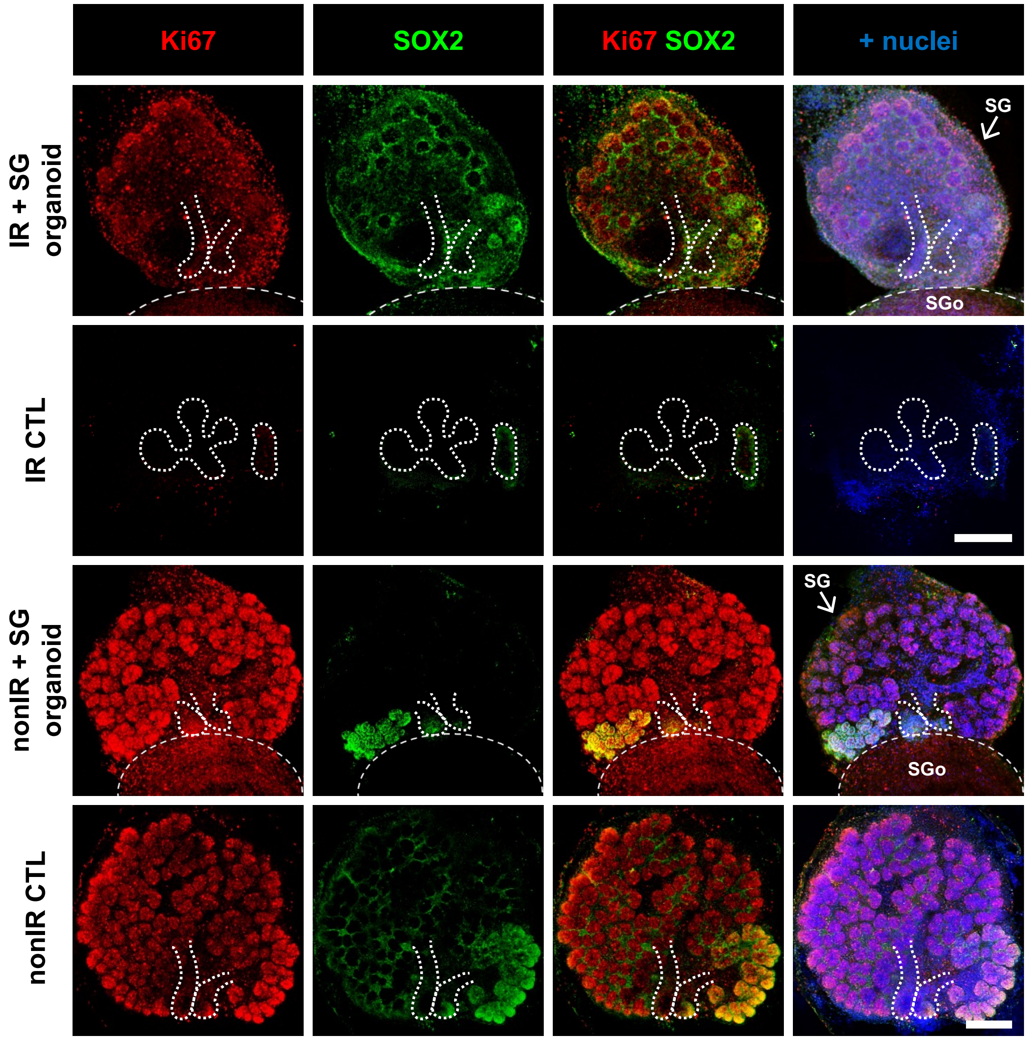

Figure S8. Cell proliferation is increased in epithelial buds and ducts after transplantation of SG-like organoids in both irradiated and non-irradiated SG models. SG-like organoids were defined as controls for this study. Representative maximum intensity images of mouse SG of each group immunostained for proliferative cells (Ki67), SG progenitors (SOX2) and counterstained with a nuclear dye. Dotted white line denotes the SG ducts. Scale bar: 200 µm. SGo: salivary gland organoid. IR: irradiated. nonIR: non-irradiated.

Figure S9.

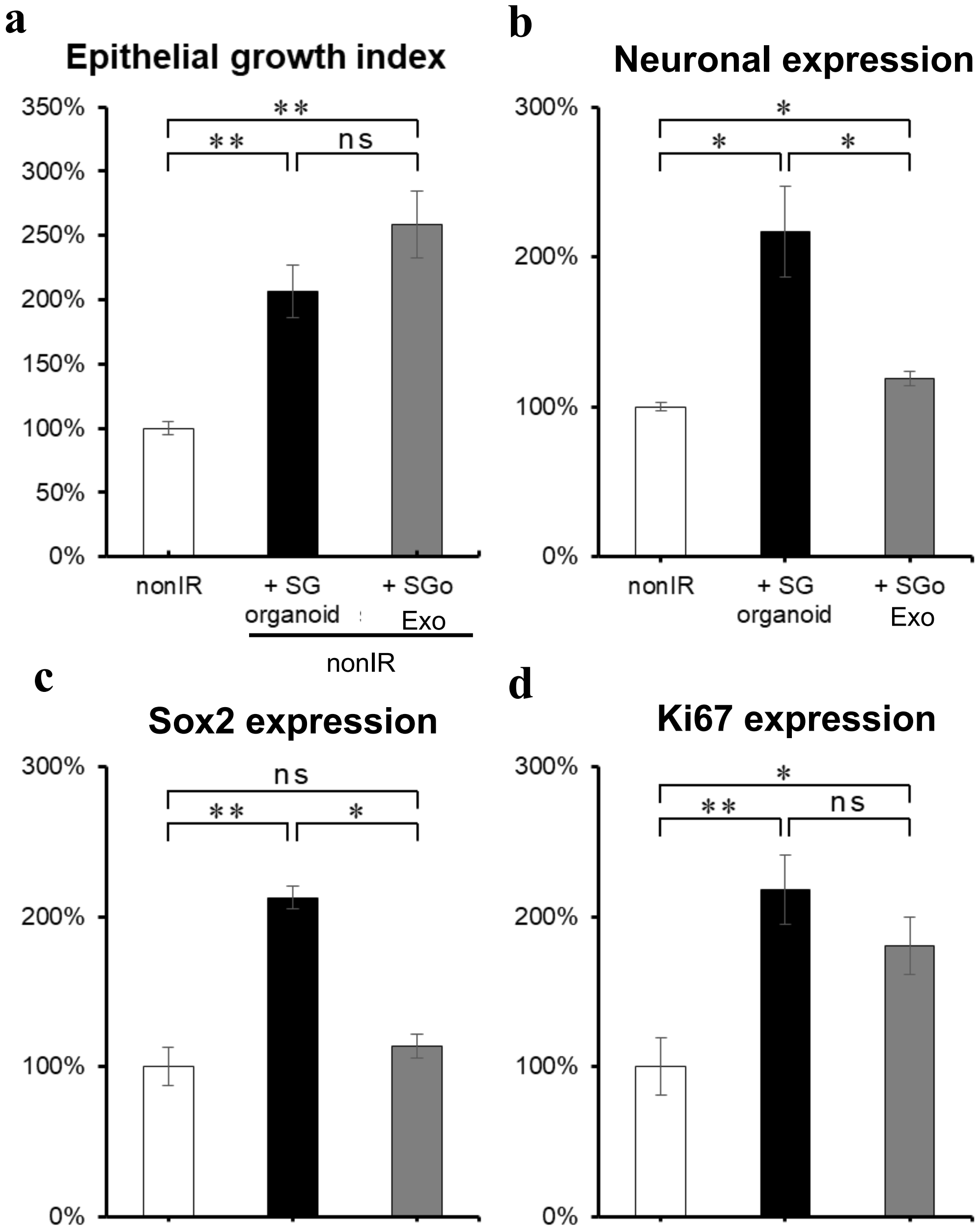

Figure S9. Epithelial growth (a) and expression of neurons (b), mitotic markers (c), and SG stem/progenitor marker (d) in normal nonIR SG models treated with SG-like organoids or with SGo exosomes. The epithelial growth index was calculated based on the Spooner's ratio and was divided by the ratio of the nonIR control group. The protein expression were determined after immunofluorescence staining using Imaris software analysis in both glands (submandibular and sublingual). This protein expression data was calculated relative to nonIR SG expression, and values were normalized to total explant or nuclear area (as demarcated by the Hoechst 33342 nuclear staining). Data are presented as mean ±SEM (n = 5-6). ns: no statistical difference, *p<0.05, **p<0.01, ****p<0.0001. SGo: salivary gland-like organoid, nonIR: non-irradiated.
